# Betting, Forex Trading, and Fantasy Gaming Sponsorships—a Responsible Marketing Inquiry into the ‘Gamblification’ of English Football

**DOI:** 10.1007/s11469-017-9788-1

**Published:** 2017-07-25

**Authors:** Hibai Lopez-Gonzalez, Mark D. Griffiths

**Affiliations:** 1 International Gaming Research Unit, Psychology Division, Nottingham Trent University, 50 Shakespeare Street, Nottingham, NG1 4FQ UK; 2 Psychology Department, University of Deusto, Bilbao, Spain

**Keywords:** Gambling, Sports betting, Marketing, Advertising, Forex trading, Fantasy gaming

## Abstract

Environmental stimuli in the form of marketing inducements to gamble money on sports have increased in recent years. The purpose of the present paper is to tackle the extended definition of the gamblification of sport using sponsorship and partnership deals of gambling, forex trading, and fantasy gaming as a proxy for assessing its environmental impact. Using data about sponsorship deals from English Football Premier League, the paper builds on the evidence of English football’s gamblification process to discuss the impact that the volume, penetration, and marketing strategies of sports betting might have on public health and well-being. Findings demonstrate that gambling marketing has become firmly embedded in the financial practices of many Premiership football clubs. It is argued that such associations are not trivial, and that the symbolic linkage of sport and newer gambling forms can become an issue of public health, especially affecting vulnerable groups such as minors and problem gamblers. The present study is the first to explore in-depth the relationship and potential consequences and psychosocial impacts of sports-related marketing, particularly in relation to football.

Environmental stimuli in the form of marketing inducements to gamble money on sports have increased in recent years (Danson 2010), with numerous social groups and scholars raising concerns about the unknown long-term effects of gambling marketing (e.g. Thomas et al. 2012a; Yani-de-Soriano et al. 2012). Although accounts of bookmakers directly influencing the formal codification of the laws of sport date back to the 18th century (Forrest and Simmons 2003), there is a growing consensus that a sizeable part of the world today is witnessing an unprecedented proliferation of gambling products focusing on the outcomes of sports competitions (Dyall et al. 2009; Hing 2014; LaPlante et al. 2008; McKelvey 2004; Sproston et al. 2015). Such proliferation prompted McMullan and Miller (2008). As the *-fication* suffix implies, gamblification is a process that of expanding the areas of sport susceptible to being gamble upon, which has been further accelerated by the nearly simultaneous legalisation and popularisation of Internet gambling in different jurisdictions (Gainsbury et al. 2012).

In the decade since the gamblification of sport theory was proposed, the term has evolved from its original meaning. Sport gamblification is no longer restricted by the limits of sports betting and sport per se. On the contrary, it has developed multiple interactions with adjacent markets. Aligning with a broader context of technological integration of gambling products, as seen in the convergence of gambling and social gaming (Cassidy 2013; (Griffiths et al. 2014), casino gaming (Gainsbury et al. 2014), and digital media (King et al. 2010), sports betting is also under its own process of integration. Sports betting convergences—most notably with trading markets, such as foreign exchange (forex or FX), poker, and daily fantasy games (Lopez-Gonzalez and Griffiths 2016b)—have been further facilitated by the evolution of big data retrieval from sports events (Hutchins 2016). Such developments in sports data retrieval include traditional visual methods, as well as newer motion-tracking cameras, smart sensors embedded in equipment, wearables, and body response tracking devices (Marr 2015). Sport organisations have capitalised on the convergence dynamic by signing formal financial agreements with gambling firms (Hing et al. 2017; Hing et al. 2014; Lopez-Gonzalez and Tulloch 2015; Sproston et al. 2015), but their involvement with the extended definition of gambling markets, including gambling-like products such as trading markets and fantasy games, remains largely unexplored. The convergence of gambling, trading, and gaming markets calls for such an extended understanding of what constitutes gambling today, particularly in the way gambling harm-minimisation and responsible marketing campaigns are designed.

A major preoccupation regarding gambling intersection with sports has been the marketing of betting as an experience inherently associated with the symbolic culture of sport (Deans et al. 2016, 2017a; Gordon and Chapman 2014; Lamont et al. 2011; Thomas et al. 2015). By emphasising its connections with sports, the marketing and advertising of betting has been theorised to pursue the ‘sanitation’ of gambling (McMullan and Miller 2008), transferring the health-related symbolic attributes of sport and physical exercise to betting behaviour (Milner et al. 2013). In this regard, and of great concern, is the effect that an excessive volume of betting marketing might have on vulnerable groups such as minors and young adults (Pitt et al. 2016; Thomas et al. 2016), and individuals suffering or recovering from gambling problems (Hing et al. 2015, 2016b). Furthermore, additional issues might arise in the event that these new categories that extend the definition of sports gambling (i.e. trading, other gambling forms such as poker, and fantasy games) seeking to market their products in alignment with (or appropriation of) sports’ core values and positive attributes. Early examples of this marketing strategy can be found in the sport stars’ endorsement of poker brands such as the footballers Neymar Jr. and Cristiano Ronaldo, and the tennis player Rafael Nadal (Lopez-Gonzalez and Griffiths 2016b).

The present paper tackles the extended definition of the gamblification of sport by using sponsorship and partnership deals of gambling, forex trading, and fantasy gaming as a proxy for assessing their environmental impact. Using data from English Football Premier League, the paper builds on the evidence of English football’s gamblification process to discuss the impact that the volume, penetration, and marketing strategies of sports betting might have on public health and wellbeing. The study is constructed in the form of a conceptual paper that (i) explores the legal framework and (ii) marketing evolution of gambling in English football, (iii) combining it with new descriptive evidence from sponsorship and partnership deals, to finally (iv) speculate on the detrimental consequences that such marketing might have, based on the existing literature on betting marketing-related harm.

## Sports Betting Marketing in the UK

### Legal Framework

Gambling operators can legally market online and offline products in the UK provided they are licensed to do so by the Gambling Commission, the regulatory body created after the Gambling Act 2005. The gambling industry regards the UK as the most developed gambling regulatory system in Europe (Danson 2010). Some scholars have argued that gambling, despite having been a well-established and culturally sanctioned practice for centuries in Britain (Forrest and Simmons 2003; Schwartz 2006), was presented for the first time to the British public by the Gambling Act as a ‘viable and socially acceptable leisure activity’ (Parke et al. 2014, p. 24). However, gambling is still regarded—at least in social and economic terms—as a demerit good, in the same vein as tobacco or alcohol, and therefore subject to above average taxation in an attempt to counterbalance the negative effects of its consumption. Demerit goods are those goods that are widely perceived as ‘intrinsically unhealthy, degrading or socially damaging’ (McCormick and Stone 2007, p. 162). Such mixed understanding of gambling as a legal, tax-collecting, but at the same time demerit activity transpires in the law in the form of regulatory requirements that some scholars have come to label as hypocritical (Myllymaa 2017).

The responsibility of controlling sports betting marketing and advertising in the UK lies on a multi-node network operating under the Gambling Act 2005 and the Gambling (Licensing and Advertising) Act 2014 (Lopez-Gonzalez and Griffiths 2016a). The Gambling Commission, as noted above, is the regulatory body responsible for issuing licenses and regulating gambling activity. Also, in terms of an advertising activity, gambling marketing is further controlled by the Advertising Standards Authority (ASA), which oversees the application of both the UK Code of Broadcast Advertising, and the UK Code of Non-Broadcast Advertising. Moreover, the Office of Communications (Ofcom) protects British consumers from promotions that could be harmful or offensive. Additionally, the gambling industry complements the existing legal framework with self-regulatory codes.

Generally speaking, the UK Gambling Commission has shown specific concern regarding two aspects of betting marketing: the promotion of the so-called free bets and children’s protection. The Gambling Commission stipulates that free bet promotions must include *in the advert itself* any condition or factor that might affect the bets, such as withdrawal requirements. This follows up on the ASA guidance, in response to an increasing number of complaints by bettors concerning the misleading terms of many gambling products advertised as risk-free or simply free (Committee of Advertising Practice and Broadcast Committee of Advertising Practice 2014).

Sports betting marketing cannot target minors and, in general, cannot feature in their promotions anyone who is or appears to be under 25 years old (Advertising Standards Authority 2014), although some exceptions apply to betting websites featuring sportspeople after the operators’ complaints regarding the prohibition to use the image of the footballer Luis Suárez (24 years old at the time) and the golfer Jordan Spieth (aged 22 years at the time) (Smith 2016). Given the rise of foreign gambling operators following sport sponsorship marketing strategies in British football, the Gambling Commission released a one-page advice note in November 2014 to clarify its position on it. The advice solely focused on child protection, reminding consumers that according to the Gambling Act 2005, ‘gambling operators will not allow their logos or other promotional material to appear on any commercial merchandising which is designed for use by children. A clear example of this would be the use of gambling logos on children’s football strips’ (Gambling Commission 2014).

Sport and gambling organisations materialise their shared interests by signing partnership and sponsorship deals, either at competition level (i.e. leagues), organisational level (teams), and/or individual level (sport stars). On top of the financial return agreed on such deals, some sports have claimed additional compensation from betting firms on the grounds of an alleged ‘right to consent to bet’ by virtue of which sport events should be considered proprietary content, with an *authorship*, and therefore subject to copyright protection. In French sport, where this rule has been enforced, sports organisers with fixtures covered by bookmakers received 1.1% of the bets placed by legal operators in the country from 2010 to 2012 (Autorité de régulation des jeux en ligne [ARJEL] 2013), although a 2014 investigation on the matter appointed by the European Commission found little basis for the application of the right to consent to bet (Asser Institute 2014). However, beyond the legal standpoint, sport competitions have arguably few incentives to pursue legal action and be at odds with gambling industry, considering the lucrative sponsorship and partnership deals that a profitable relationship with betting firms has brought to them. Trading and fantasy gaming partnerships with sports industry have not attracted similar public scrutiny and there is no provision that specifically addresses their use in the context of sports betting marketing and advertising.

### Volume

Marketing and advertising expenditure growth in the UK mirrors that of several European and overseas countries that—since the legalisation of online gambling in early 2010s—have witnessed a surge of gambling inducements (European Commission 2012). Increased marketing following global online gambling adoption has been visible both in territories with a relatively small gambling culture such as Spain (Dirección General de Ordenación del Juego [DGOJ] 2017 [Directorate General of Gambling Regulation DGOJ]), as well as in well-established mature markets like Australia, where online sports betting has become the only gambling form with an expanding customer base over the last decade (Gainsbury et al. 2015).

In 2012, sports betting adverts accounted for approximately 0.3% of all commercial adverts a British adult saw, with almost half of them (45.2%) being seen while watching sports. In a 1-year period, from 2011 to 2012, betting adverts in the UK rose by 270%. Children aged 10–15 years (who are supposedly protected by not seeing gambling adverts on television before 9 p.m.) were reached 148,000 times throughout 2012, 50 times the volume of 2005, because of the legal loophole that allows betting adverts to be broadcast during televised sporting events (Office of Communications 2013). A journalistic report by Nielsen commissioned by *The Guardian* newspaper in the UK calculated that betting companies had spent £456 million on British television adverts from 2012 to 2015, with an annual growth of 46% (Davies 2016). Although the causal link between advertising expenditure and gambling engagement is not easy to establish, industry data provided by the Gambling Commission suggests a parallel evolution (Gambling Commission 2017). For example, when considering the (non-remote) betting gross yield (i.e. the money bookmakers retain after paying out prizes for bets won), the UK betting market had a size of £3029 million in 2012, £3198 million in 2013, £3176 million in 2014, £3276 in 2015, and £3406 million in 2016. This evolution represents a relatively modest 12.5% rise when compared to the 46% advertising growth over the same period. However, on top of the non-remote yield, remote (domestic) football betting in 2016 accounted for £619 million, although no historical data is available to trace back the evolution in remote betting due to new methodological arrangements in data collection (Gambling Commission 2017).

In European sporting leagues as a whole, shirt sponsorships in the top five football markets (i.e. England, Germany, France, Italy, and Spain) grew from only one deal in 2002/03 season to 26 deals in the 2010/11 season (Foley-Train 2014). According to latest free-access data from the European Sponsorship Association reported by Foley-Train, gambling operators ranked seventh of all business sectors for worldwide sport sponsorship deals in 2012 (74 deals), tripling the deals made in 2007 (21 deals). For the UK in particular, industry data from the European Gaming and Betting Association (EGBA) estimates that betting companies in 2015 spent around €80 million exclusively sponsoring English football (EGBA 2015), almost six times more than the €14 million of annual average expenditure estimated for the 2009–2014 period (Foley-Train 2014).

### Gambling Sponsorship in English Football

Shirt sponsorship is probably a good proxy to calibrate the volume of gambling marketing in English football. Table 1 shows the shirt sponsor evolution over the past decade (from the 2007/2008 to 2016–2017 seasons). First team shirt sponsorship with gambling companies evolved from four deals in 2008, six deals in 2012, to ten deals in 2017, accounting for half of the 20 English Premier League teams. Clearly, the number of shirt logos owned by gambling brands has evolved rapidly over a relatively short period of time. However, some industry voices have been anticipating a decline in the numbers of shirts being sponsored by gambling firms due to their incapacity to compete with other business sectors (Gillooly 2015), although such a decline has yet to materialise. According to Gillooly, the average gambling sponsorship for an English Premier League club in 2014 was £3.9 million, well below the £11.2 million average for any other sponsorship in the league.

**Table 1:** Main shirt logo sponsorship deals (2008–2017) in English Premier League

	2007/2008 sponsor (team)	Industry	2011/2012 sponsor (team)	Industry	2016/2017 sponsor (team)	Industry
1	AIG (Manchester Utd)	*Insurance*	Etihad Airways (Manchester City)	Travel	Yokohama Tyres (Chelsea)	Automotive
2	Samsung (Chelsea)	Electronics	Aon (Manchester Utd)	*Insurance*	AIA (Tottenham Hot.)	*Insurance*
3	Fly Emirates (Arsenal)	Travel	Fly Emirates (Arsenal)	Travel	Etihad Airways (Manchester City)	Travel
4	Carlsberg (Liverpool)	Alcohol	Aurama (Tottenham Hot.)	Technology	Stan. Chartered (Liverpool)	*Banking*
5	Chang Beer (Everton)	Alcohol	Virgin Money (Newcastle Utd)	*Investment*	Fly Emirates (Arsenal)	Travel
6	32Red (Aston Villa)	**Gambling**	Samsung (Chelsea)	Electronics	Chevrolet (Manchester Utd)	Automotive
7	Bet24 (Blackburn Rov.)	**Gambling**	Chang Beer (Everton)	Alcohol	Chang Beer (Everton)	Alcohol
8	Oki (Portsmouth)	Electronics	Standard Charter (Liverpool)	*Banking*	Virgin Media (Southampton)	Telecom
9	Thomas Cook (Manchester City)	Travel	FXPro (Fulham)	*Trading*	Mansion (Bournemouth)	**Gambling**
10	XL Holidays (West Ham Utd)	Travel	Bodog (West Bromwich)	**Gambling**	Uk-k8 (West Bromwich)	**Gambling**
11	Mansion (Tottenham Hot.)	**Gambling**	32Red (Swansea City)	**Gambling**	Betway (West Ham Utd)	**Gambling**
12	Northern Rock (Newcastle Utd)	*Building Society*	Aviva (Norwich)	*Insurance*	King Power (Leicester City)	Travel retail
13	Garmin (Middlesbrough)	Technology	Tombola (Sunderland)	**Gambling**	Bet365 (Stoke City)	**Gambling**
14	JJB Sports (Wigan Athletic)	Sportswear	Britannia (Stoke City)	*Building Society*	Mansion (Crystal Palace)	**Gambling**
15	Boylesports (Sunderland)	**Gambling**	JJB Sports (Wigan Athletic)	Sportswear	BetEast (Swansea City)	**Gambling**
16	Reebok (Bolton Wan.)	Sportswear	Genting Casinos (Aston Villa)	**Gambling**	Dafabet (Burnley)	**Gambling**
17	LG (Fulham)	Electronics	Malaysia Airlines (Queens Park)	Travel	138.com (Watford)	**Gambling**
18	Kyocera (Reading)	Electronics	188Bet (Bolton)	**Gambling**	SportPesa (Hull City)	**Gambling**
19	F&C Investments (Birmingham Cy.)	*Investment*	Prince’s Trust (Blackburn Rov.)	Charity	Ramsdens (Middlesbrough)	*Money loan*
20	The Derbyshire (Derby Co.)	Travel	SportingBet (Wolverthampton)	**Gambling**	Dafabet (Sunderland)	**Gambling**

Teams ranked by final table positions. In bold, gambling operators. In italics, other money businesses

In the same vein, it has been noted that most of the football teams with shirts sponsored by gambling companies are among the less powerful in the league, both in terms of economic profitability and sporting success. Indeed, analysing the data from end-of-season table positions demonstrates a bias of gambling companies sponsoring teams towards the lower end of the table. Thus, the four teams (out of 20 in the English Premier League) with gambling logos in 2007/2008 finished the league in 6th, 7th, 11th, and 15th. In 2011–2012, the six teams sponsored by gambling companies finished 10th, 11th, 13th, 16th, 18th, and 20th. In 2016/2017 season, the ten teams with gambling sponsors showed an almost perfect inverse correlation between table position and gambling-origin shirt sponsor, finishing 9th, 10th, 11th, 13th, 14th, 15th, 16th, 17th, 18th, and 20th (with the 19th being a money loan company). This could be interpreted as a nuanced strategy. More specifically, gambling operators might believe they have enough global exposure that the league as whole offers, without needing to pay premium sponsorship deals to attach their brand to the most supported and successful teams (because all the lower ranked teams have to play all the upper ranked teams and therefore get equal advertising exposure during televised games).

Table 2 shows the breadth of the gamblification process by focusing on sponsorship deals during the 2016–2017 season in the English Premier League. As can be observed, all 20 teams have secured at least one official betting partner, with some of them having multiple partners due to regional deals in strategic markets to provide the so-called geo-targeted betting experiences (Garlitos 2014). An illustration example is the Arsenal club’s deals with *12Bet* company in Asia, *Betfair* in Europe, *SportPesa* in Kenya, and *Tempobet* in Oceania. Altogether, the 20 English Premier League teams totalled 20 different betting brands, with 12 brands sponsoring only one team, five brands sponsoring two teams, and three brands sponsoring three different teams. Despite how fragmented the betting market might look, these brands represent only a small fraction of the actual number operating in association with the English football. In fact, betting brands are generally considered to offer poorly differentiated products in highly competitive markets (Nettleton 2013). Consequently, marketing plays a significant part in artificially creating singular attributes that facilitate the acquisition and maintenance of customers.

**Table 2:** Gambling, trading, and fantasy games official partners and sponsors among 2016/2017 English Premier League teams

Team	Gambling	Trading	Fantasy
Arsenal	12bet, Betfair, SportPesa, TempoBet	Markets.com	DraftKings
Bournemouth	Mansion	–	–
Burnley	Dafabet	–	Sportito
Chelsea	William Hill	CWM FX^a^	–
Crystal Palace	Mansion	–	–
Everton	William Hill, Dafabet	EZTrader	Football manager
Hull City	SportPesa	–	–
Leicester City	BetStars, 12Bet	FairFX	Oulala
Liverpool	BetVictor	InstaForex	DraftKings
Manchester City	Betsafe	iTrader	Ballr
Manchester United	Marathonbet	Swissquote	–
Middlesbrough	TempoBet, TLC Bet	Ramsdens^b^	–
Southampton	SportPesa	Banc De Binary^c^	Premier Punt
Stoke City	Bet365	–	–
Sunderland	Dafabet, Betfair	Satsuma^b^	–
Swansea City	BetEast, Bet365, Swans Lotto	–	–
Tottenham Hotspur	William Hill, Fun88	EZTrader	–
West Bromwich Albion	Coral, Bet365, Uk-k8	IGOFX	–
West Ham United	Betway	eToro	–
Watford	138.com	Divisa	DraftKings, Football manager

Partners listed in the English Premier League teams’ official websites

^a^Deal was signed until 2018 but Chelsea discontinued the relationship for undisclosed reasons

^b^These are quick loan companies per se

^c^Relationship ended shortly after the onset of 2016/2017 season

Sponsorship deals with trading companies are not as prevalent as betting sponsorships. However, 14 out of 20 English Premier League teams had linked partnership deals with trading companies—most notably forex trading—during the 2016/2017 season. Only one trader (*EZTrader*) sponsors two different teams, while the rest are unique sponsors. The same betting market attributes of low product differentiation and competitive environment might also be applicable to trading firms.

Fantasy gaming is rapidly becoming a large component of sports appreciation, especially in the USA where fantasy sports appears to have partially absorbed the consumer base for online sports betting, an illegal activity in most states (Bowman et al. 2016; Rose 2015). Although still in its infancy in Europe, eight out of 20 English Premier League teams already have agreements in place with fantasy sports companies, some of which include a deal with *DraftKings*, the leading company along with *FanDuel* in USA’s fantasy gaming market. The concentration of brands here is slightly higher than in the case of betting and trading sponsorships, but six different brands still populate the growing fantasy gaming market in the English Premier League.

## Exploring the Effects of Sports Gamblification Marketing

### Does Sports Betting Marketing Facilitate Problem Gambling?

The detrimental effect on public health of an increase in the sports betting marketing volume is difficult to demonstrate. British data collected by the Gambling Commission is inconclusive due to the lack of definition of what constitutes gambling on sports. In general, research has found it difficult to substantiate the causal association between gambling advertising exposure and behaviour, particularly when the effects of such exposure might take place weeks or months later, as proposed, for instance, by the Cultivation Theory (Gerbner 1969). Despite the difficulties of finding empirical evidence of the real impact of marketing on betting behaviour, many authors have acknowledged that the association between marketing and problem gambling is plausible, at least theoretically. In this regard, Binde (2014) concluded after systematically reviewing a large body of gambling advertising literature that it is ‘very unlikely that advertising should have no impact whatsoever on problem gambling’ (p. 19). However, considering the increasing amount of money allocated to gambling advertising, it appears conceivable that British operators find an attractive return of investment to their advertising expenditure.

The sports betting marketing and advertising growth prompted by the gamblification of sport could be theorised as having two effects. First, an increase in gambling advertising exposure might be associated with a higher prevalence rate of problem gambling. A study indicated that problem gamblers are usually more exposed to advertising (e.g. they frequently visit more gambling websites or watch more sport events); therefore, it cannot be established whether they gamble more because they are more exposed to marketing or they are more exposed to marketing because they gamble more (Hing et al. 2016a). However, a study conducted among 6034 Norwegian gamblers found that problem gamblers had a greater involvement with gambling advertising even when they were similarly exposed than regular non-problem gamblers (Hanss et al. 2015). It is worth noting that available data from the UK shows no indication that gambling advertising has led to more gambling harm. For the period of greatest gambling advertising growth (2012–2015), the problem gambling prevalence rate in Scotland stayed relatively stable, moving from 0.8% in 2012 to 0.7% in 2015 (Gambling Commission 2016). The latest data from England dates back to 2012; therefore, the impact (if any) on public health of online betting adoption due to the rise in gambling advertising is as yet unknown.

Second, an overall rise in the consumption of gambling products following more aggressive marketing strategies, even while maintaining a stable percentage of people experiencing gambling-related harm, would lead to a rise in absolute numbers of people developing gambling problems (Hansen and Rossow 2012). Simply put, keeping problem gambling rate constant, the more people that bet on sports, the more problem gamblers. This is known as the total consumption model. Some scholars have criticised this model by arguing that sports betting marketing does not necessarily create new consumers, and that it only affects market share, transferring existing bettors from one bookmaker to another (Hing 2014). The gambling operators’ awareness of the inherent growth limits of the British market could help to interpret the recent mergers between large gambling firms in the UK as an attempt to minimise the huge costs of an intensive competition prolonged over time. Other scholars support social adaptation models (e.g. Shaffer et al. 2004) that propose that while the detrimental effects of marketing and advertising might be true in the initial stages of the exposure, individuals and societies adapt their attitudes, cognitions, and behaviours in the long term, generating resistance to the exposure, for example, by enhancing the social awareness of the problem, counter-advertising the industry’s messages, setting up prevention programmes and treatment units, or simply the novelty of the products wearing off as time goes by.

### The Naturalisation Effect

Departing from the impact that a larger volume of gambling stimuli might have on individuals developing gambling problems (as well considering the complexity of assessing marketing’s direct effects), other indirect effects that have an impact at group level can be posited. Some of the most notable aspects of gambling marketing are its ubiquity and penetration. Gamblers have repeatedly described commercial stimuli to bet as being everywhere (Pitt et al. 2016). In this regard, the specific messages or narratives that are conveyed by such stimuli do not appear to be as crucial as the accumulative presence of the stimuli. Although each individual brand emphasises their own singular value to their potential target customers, taken as a whole, the aggregated message is more likely to be interpreted along the lines of ‘we are here, we exist’. The corollary of this being the real core marketing message of the betting industry has prompted some scholars to conduct scans to determine the ‘environmental impact’ that the accumulation of betting stimuli might have on the society as a whole (e.g. Sproston et al. 2015).

Some authors have examined the outreach of specific brands, describing how they structure traditional and social media channels to position their products in every market (Sproston et al. 2015). Other studies have examined the combined gambling impact an individual views during in an individual sport event. In the Australian National Rugby League (NRL) in 2012, a spectator watching a game was targeted an average of 322 times with gambling stimuli (Lindsay et al. 2013). Today, gambling stimuli in sports broadcasts, especially football, include live match commentary, sponsors of the networks with onset promotions, commercial breaks before, after and during half-time, electronic and physical banners around the field, shirt logos, on-screen live odds, as well as projected holograms and augmented reality messaging onto the venue’s pitch (Lopez-Gonzalez et al. 2017).

The aggregation of marketing strategies over long periods of time has raised concerns regarding the naturalisation or normalisation of gambling on sports over the long term (Deans et al. 2017a, b; Gainsbury and Russell 2015; Hing et al. 2016a; Lamont et al. 2011; Sproston et al. 2015). The naturalisation effect is especially concerning in the case of children and adolescents, but also in the case of young adults, with insufficient historical perspective to compare the current betting context against previous periods where the union of sport and gambling was not so pervasively emphasised by betting marketers. Bettors’ data directly extracted from gambling companies in Germany indicates that the average age of regular bettors is becoming lower (Gassmann et al. 2015). In that regard, experiments with children aged 5 to 12 years have demonstrated how prevalent their recognition of betting sponsors in association with sport teams is, to the point of recalling sponsorships that never took place (Bestman et al. 2015). Other forms of gambling have prompted children to think in the past that gambling can be a tool for self-expression and rebelliousness (Korn et al. 2005). If the association between gambling and sport becomes fully naturalised, gambling is likely to integrate among its core components the perceived positive attributes of contemporary sport. Here, the present authors provide a tentative list of some of these components:

(i) The role of those practicing sport is active, their actions being the ones determining the outcome of any game.
(ii) Sport is based on talent. The role of chance is inversely proportional to the amount of skill teams or individuals can achieve.
(iii) Sport is based on preparation and work. It is a practice that can be mastered with sufficient training, self-commitment, and discipline (Milner et al. 2013).
(iv) Experience and knowledge are instrumental to success.
(v) Being merit-based, those who have not succeeded have only themselves to blame, considering the equal opportunities the system allows.
(vi) Sport stars represent the story of successful risk-takers that bet against the odds, given the unlikelihood of becoming a professional player, but prevailed in the end.
(vii) For those who excel at it, sport is a very profitable activity.
(viii) Sport is a manifestation of masculinity and control (Anderson 2009; Whannel 2001).
(ix) Sport is fun and exciting.
(x) Sport is safe, harmless, and promotes healthy behaviours (McMullan and Miller 2008).

The following section explores the potential dangers of the transference of meaning from each sport characteristic listed above into the remit of gambling, trading, and fantasy gaming.

### Sporting Attributes in Trading and Gaming

(*i*, *ii*, *iii*, *iv*, and *v*): Online betting marketing represents a shift of focus from the traditional view of weekly football pools as a rather passive gambling form where bettors would typically fill out a column with the expected winners of the fixtures of a Premier League week and then wait until the events unfolded. In stark contrast, contemporary betting marketing highlights the active role of bettors and the way mobile technologies can be used to enhance the control over the betting experience, and implicitly, over the bet outcome itself (Lopez-Gonzalez et al. 2017).

The development of betting interfaces towards more involved experiences featuring more options for bettors to interact with the ongoing bets (e.g. ‘cash out’) has *gamified* sports betting (Lopez-Gonzalez et al. 2017). This development might punctuate a transition towards a more comprehensive convergence of betting and gaming businesses. If that occurs, the line between the perceived control over the platforms and interfaces, and the actual control over the bets placed will be blurred in bettors’ minds, resulting in less planned betting behaviour.

The more activated the role of bettors is meaningful in terms of and enhanced ‘illusion of control’ (Langer 1975) and overconfidence over one’s own skills (Svenson 1981). Furthermore, it also brings to life the idea that in sports betting, training, knowledge, experience, and skills separate those who win from those who lose. Betting marketing emphasises the idea that all the relevant data to inform bets is accessible for bettors, either in the form of data, statistics, or media reports. It follows that betting is a rational activity that comprises the analysis of available information that, if done diligently, yields profits. Fantasy gaming is a good example of this. Fantasy sports are legal in the USA, despite the prohibition of online gambling in the country, because companies have been successful (to date) in arguing at court cases that their products are based on acts of skill, not chance (Rose 2015). Similarly, trading is also based on the assumption that the chance component can be mastered with enough training and experience. Marketing campaigns overestimate the role of skill (while underestimating the importance of luck), encouraging gamblers to persevere in their skill acquisition. This is relevant in the case of children, because their cognitive skills are still developing, and problem gamblers, who often have distortions and biases in their cognitive processing (Ciccarelli et al. 2017; Griffiths 1994; Parke and Griffiths 2012).

(*vi*, *vii*, and *viii*): Conceptually speaking, one of the characteristics that distinguishes a trader from a gambler is professionalism. Being a trader is perceived to be a job, and it is defined in the Cambridge Dictionary (2017) as a person who buys or sells company shares or money, whereas in the same dictionary, a gambler is defined as a person who risks money. The juxtaposition of gambling and trading, by means of sport, is likely to transfer some of the connotations of professional, business-oriented task of trading to the gambling realm. This juxtaposition is furthered by sport stars, who also add the business component to something that was primarily meant to be an entertainment.

Trading borrows from sport the attributes of speed, immediacy, precision, and technological innovation, which is why Formula 1 car racing has been an ideal target competition for forex companies (Pearson 2013). The preponderance of those same attributes of speed and precision could also help to explain the selection of Usain Bolt, the eight-time Olympic gold medallist sprinter, as a brand ambassador for the *XM Forex* company in 2016 (Dudley 2016). The symbolic association of gambling and trading via sports makes gambling depart even further from being considered a game of chance, focusing on the components of skill, knowledge, and analysis.

Trading is likely to be an environment of extreme competition, which translates into greater risk of losing the invested money. However, in contrast to such negative characterisation, trading marketing via sports, frames competition as positive, involving a symbolic understanding of trading that entails elements such as the survival of the fittest, the thrill of taking risks, while curtailing any moral obligations caused by irresponsible trading. In close relation to this, the building up of masculinity has therefore been proposed as a mechanism used by gambling advertising to induce riskier betting behaviour (Thomas et al. 2012b). Arguably, trading also builds on recklessness (positively framed as controlled risk-taking) to construct traders and gamblers to the image of sports stars, borrowing characteristics from them such as instinctive behaviour, decisiveness, and on-the-go decision-making. Recent research in the UK has shown that bookmakers manage greater profit margins when presenting bettors with multiple outcome bets whose risk and winning probability is hard to calculate accurately (Newall 2015, 2017).

In addition, elite football evokes a life of affluence. Sport stars often represent stories of working class people who become millionaires almost overnight, typically in their early 20s (Smart 2005). In that sense, sport enacts narratives of easy money and strikes of luck, and an association in the mind of quick money. The contention here is that the extended gambling definition proposed in this paper does not happen in isolation, and that the convergence of gambling, gaming, and trading businesses is more likely to evolve in an atmosphere saturated by other inducements to play with money. Looking again at Table 1, gambling and trading shirt sponsors align next to brands that could be similarly construed as money businesses: building societies (*Northern Rock*, *Britannia*), investment firms (*F&C Investments*, *Virgin Money*), insurance companies (*AIG*, *Aviva*, *Aon*, *AIA*), and a quick loan company (*Ramsdens*). In total, the number of companies inducing consumers to pay now and expect greater return in the future accounted for seven sponsors in 2007/2008, 11 in 2011/2012, and 12 in 2016/2017, embedding further the overall sensation of sport gamblification.

(*ix* and *x*): As mentioned at the beginning of the present paper, gambling products (and even more so trading products) are widely perceived as demerit goods from an economic standpoint (McCormick and Stone 2007). However, unlike other forms of gambling, sport confers betting the attributes of healthiness, safety, and harmlessness (McMullan and Miller 2008). This association is reinforced inasmuch as sports betting becomes more gamified, symbolically departing from more obscure gambling connotations into areas of social perception characterised by playfulness and entertainment. Playing is (by definition) non-serious and entails a world of reparable consequences, as the betting marketing of free bets and guaranteed money-back offers appear to imply. The gamification of gambling (Owens 2012) adds to the active role of bettors, enhancing their perception of control over the outcome of their bets, with fantasy gaming being a by-product of such evolution.

An interpretation of the marketing rationale for the alliance of forex trading companies with sport organisations is the strategy of transferring ludic components from sports domains to re-frame trading as a harmless and entertaining activity. Take as an illustrative example the following inducement made by *FX Primus*, trading partner of English Premier League football club Manchester City until 2016, as advertised in their website:

[*Headline*] A fusion of Forex and football. [*Text*] FX trading and football might have more in common than you think…For those of you who enjoy the EXCITEMENT [*capital letters in the original*], SUSPENSE and SPEED of the beautiful game, then you have already experienced the THRILL that our traders experience when they make money on a trade (Richardson 2015).

This short inducement summarises many of the points made already in the present paper and exemplifies what irresponsible marketing of trading might look like. First, it relies on the ‘trading is like a sport’ metaphor, borrowing the component of fun and excitement from it. Second, it presents trading as an activity that has no (negative) consequences, in which people engage just for the sake of fun. Third, it draws on the emotional relationship of fans with their sports and teams to evoke a similar feeling in trading.

## Concluding Remarks

The present paper has argued for an extended definition of sport gamblification, exploring the relatively new phenomena of forex trading and fantasy gaming as an expansion of the remit of sports gambling. Using the English Football Premier League as a case study, the paper examined the potential harm involved in the association of sport with gambling and its adjacent industries (i.e. forex trading, fantasy gaming). There is a wide consensus that sports betting marketing (and advertising) must be regulated, and is the case in most jurisdictions including the UK. However, there is no specific protection concerning the marketing of trading and fantasy gaming as a specific product category associated with sports. Here, it has been contended that such association is not trivial, and that the symbolic linkage of sport and these newer gambling forms can become an issue of public health, especially affecting vulnerable groups such as minors and problem gamblers.

The present paper explored shirt sponsorship and official partnerships as a proxy for assessing the volume and penetration of sports gamblification. Although there is no scientific evidence that these marketing agreements are actually having a detrimental effect on the aforementioned vulnerable groups, it has been argued that it makes theoretical sense to think that they might potentially cause harm among some individuals. Consequently, the current online gambling restrictions should expand, in the same vein as the definition of gambling, to protect consumers from irresponsible marketing in general and the inadequate association of sport and gambling in particular.
